# Regulatory mechanisms of immune checkpoints PD-L1 and CTLA-4 in cancer

**DOI:** 10.1186/s13046-021-01987-7

**Published:** 2021-06-04

**Authors:** Hao Zhang, Ziyu Dai, Wantao Wu, Zeyu Wang, Nan Zhang, Liyang Zhang, Wen-Jing Zeng, Zhixiong Liu, Quan Cheng

**Affiliations:** 1 Department of Neurosurgery, Xiangya Hospital, Central South University, Changsha, China; 2 Department of Oncology, Xiangya Hospital, Central South University, Changsha, China; 3 One-third Lab, College of Bioinformatics Science and Technology, Harbin Medical University, Harbin, China; 4 Department of Pharmacy, Xiangya Hospital, Central South University, Changsha, China; 5 National Clinical Research Center for Geriatric Disorders, Xiangya Hospital, Central South University, Changsha, China

**Keywords:** PD-L1, CTLA-4, Cancer immunotherapy, Regulatory mechanism, Drug intervention

## Abstract

The cytotoxic T-lymphocyte–associated antigen 4 (CTLA-4)/B7 and programmed death 1 (PD-1)/ programmed cell death-ligand 1 (PD-L1) are two most representative immune checkpoint pathways, which negatively regulate T cell immune function during different phases of T-cell activation. Inhibitors targeting CTLA-4/B7 and PD1/PD-L1 pathways have revolutionized immunotherapies for numerous cancer types. Although the combined anti-CTLA-4/B7 and anti-PD1/PD-L1 therapy has demonstrated promising clinical efficacy, only a small percentage of patients receiving anti-CTLA-4/B7 or anti-PD1/PD-L1 therapy experienced prolonged survival. Regulation of the expression of PD-L1 and CTLA-4 significantly impacts the treatment effect. Understanding the in-depth mechanisms and interplays of PD-L1 and CTLA-4 could help identify patients with better immunotherapy responses and promote their clinical care. In this review, regulation of PD-L1 and CTLA-4 is discussed at the levels of DNA, RNA, and proteins, as well as indirect regulation of biomarkers, localization within the cell, and drugs. Specifically, some potential drugs have been developed to regulate PD-L1 and CTLA-4 expressions with high efficiency.

**Supplementary Information:**

The online version contains supplementary material available at 10.1186/s13046-021-01987-7.

## Background

Immunotherapy, mediated by immune checkpoint inhibitor (ICI), represents a turning point in the anti-tumor treatment of various cancer types in recent years. So far, several immune checkpoint targets, like T cell immunoglobulin and mucin-domain containing-3 (TIM-3), lymphocyte activation gene-3 (LAG-3), T cell immunoglobulin and ITIM domain (TIGIT), indoleamine 2, 3-dioxygenase 1 (IDO1), and V-domain immunoglobulin suppressor of T cell activation (VISTA), have been identified [1, 2], for which ICIs were designed and have presented promising results (Table S1). However, only ICIs targeting cytotoxic T lymphocyte antigen 4 (CTLA-4) and programmed cell death protein 1 (PD-1)/ programmed cell death-ligand 1 (PD-L1) have been FDA-approved and widely used. CTLA-4 is a classical immune checkpoint molecule, acting as a CD28 homolog with a stronger binding affinity to its receptor B7-1 (CD80) or B7-2 (CD86). As the first available ICI, the humanized CTLA-4 antibody ipilimumab has revolutionized clinical cancer care and prolonged the 10-year survival for metastatic melanoma [3]. PD-1 is encoded by PDCD1, and its ligands, PD-L1 and PD-L2, are encoded by CD274 and CD273, respectively. Overall, PD-1/ PD-L1 axis has been more widely explored and applied than PD-1/ PD-L2 axis. CTLA-4/B7 and PD-1/ PD-L1 axis regulate physiological immune homeostasis, downregulate inflammatory responses, and presumptively facilitate immune evasion of cancer cells [4–6]. Blockade of PD-1/ PD-L1 axis and CTLA-4/B7 axis promoted overall survival in multiple cancer types with significantly increased response rates of therapy [3, 7]. Blockade of PD-1/ PD-L1 axis had decreased side effects compared with blockage of CTLA-4 [8, 9]. Notably, antibodies blocking the PD-1/PD-L1 axis have been approved as first-line or second-line treatment modalities in a wide range of cancers [6, 10]. Further, combined anti-CTLA-4/B7 and anti-PD1/PD-L1 therapy has been applied to cancer treatment [11]. Nevertheless, despite promising clinical management results, only a minority of patients receiving ICIs targeting PD-L1 or CTLA-4 experience longer overall survival. Besides, ICIs failed to show significant therapeutic benefit in clinical trials of colorectal carcinoma (CRC) (confirmed responses in 7 of 84 patients), non-small cell lung cancer (NSCLC) (confirmed responses in 5 of 28 patients), melanoma (confirmed responses in 9 of 22 patients), and glioblastoma (confirmed response rate: 23.1%) [12–14].

**Fig. 1:**
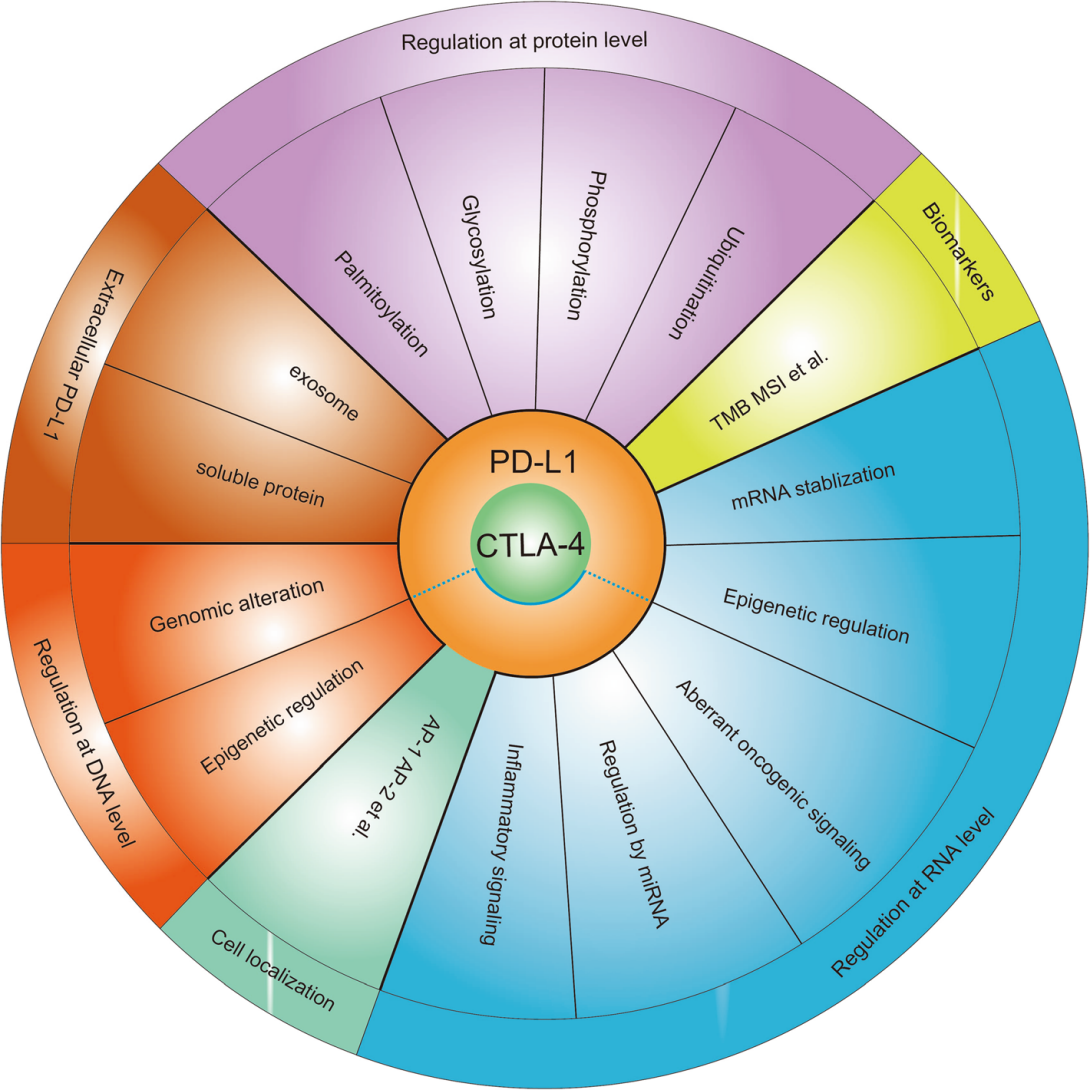
Overall regulatory mechanisms of PD-L1 and CTLA-4. TMB, tumor mutation burden; MSI, microsatellite instability; AP-1, adaptor protein 1; AP-2, adaptor protein 2.

CTLA-4, primarily expressed by T cells, binds to CD80/CD86 and prevents the stimulatory signaling of T cell proliferation provided by binding of CD28 with CD80/CD86 during the priming phase [15]. In contrast, the immune evasion process led by PD-1/ PD-L1 axis is primarily attributed to the overexpressed PD-L1 on cancer cells which binds to PD-1 expressed on antigen-stimulated T cells and inhibited the activity of PI3K/AKT and Ras/MEK/ERK signaling pathways. Consequently, this impairs the proliferation, differentiation, and activation of T cells during the effector phase [16]. Besides tumor cells, PD-L1 could be expressed on tumor-infiltrating lymphocytes (TILs), including tumor-associated macrophages (TAMs), dendritic cells (DCs), regulatory T cells (Tregs), cancer-associated fibroblasts (CAFs), mast cells, and tumor stroma cells (astrocytes, vascular endothelial cells, keratinocytes) [4]. Therefore, PD-L1 overexpression on tumor cells, TILs, or tumor stroma cells inevitably triggers T cell exhaustion, thereby creating an immunosuppressive microenvironment and promotes tumor progression [17]. Correspondingly, PD-L1 amplification has commonly been used as a positive predictive biomarker to blockade the PD-1/ PD-L1 axis in malignancies, including melanoma, NSCLC, and bladder cancer [4, 18, 19]. Multiple regulators influence PD-L1 expression at DNA, RNA, protein level, and drug intervention via direct regulation. Further, the expression level of PD-L1 is predicted and indirectly regulated by different biomarkers. Similarly, CTLA-4 expression, specifically regulated by its localization within the cell, regulates anti-cancer responses [6]. Therefore, a comprehensive summary of PD-L1 and CTLA-4 regulators and their common regulatory mechanisms could help identify patients with favorable response to anti-PD-L1 and anti-CTLA-4 treatments and promote their clinical management (Fig. 1).

## Searching strategy

The gene regulation can be roughly categorized into three levels including transcription level, post-transcription level, and post-translation level. At different levels of gene regulation, there are different classical regulators. Therefore, we first designed a frame structure for PD-L1 and CTLA-4 regulation at three levels. Then, we carefully searched for the relevant references from PubMed illustrating PD-L1 regulation or CTLA-4 regulation from different perspectives using the following keywords such as inflammatory signaling (IL6, TGF-b, IFN-I), oncogenic signaling (EGFR, ERK, STAT), epigenic modifier, and protein modification (ubiquitination) et.al. We also tried to review the novel points of PD-L1 regulation or CTLA-4 regulation using the keywords such as exosome and cell localization. Finally, we systematically searched for the literatures illustrating drugs or compounds that target PD-L1 or CTLA-4 and influence the expression of these two genes.

## Regulation of PD-L1 and CTLA-4 at DNA level

### Genomic alterations at the CD274 locus

**Fig. 2:**
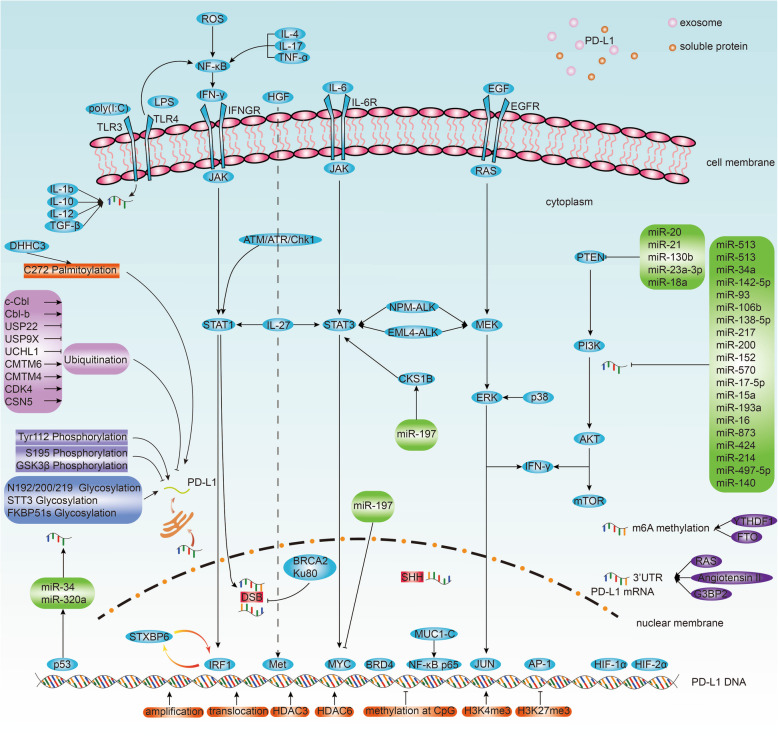
Regulatory mechanisms of PD-L1. PD-L1 expression is regulated at RNA level (inflammatory signaling, aberrant oncogenic signaling, regulation by miRNA, epigenetic regulation, mRNA stabilization), DNA level (genomic alteration, epigenetic regulation), and protein level (ubiquitination, phosphorylation, glycosylation, palmitoylation). Extracellular PD-L1 (exosome, soluble protein) and biomarkers also regulate PD-L1 expression.

CD274 is located on chromosome 9p24.1, where amplifications and translocations are linked to upregulated expression of PD-L1 in Hodgkin's lymphoma [20, 21], small cell lung cancer (SCLC) [22], NSCLC [23], lymphoma [24], Epstein-Barr virus (EBV)-positive gastric cancer [25], and oral squamous cell carcinoma (OSCC) [26]. Notably, in SCLC, CD274 amplification is caused by chromosome rearrangements without affecting the open reading frame [22] (Fig. 2, Table 1).

**Table 1:** Regulators of PD-L1

Regulatory Types	Regulators		PD-L1	Cancer types		References
**Regulation of PD-L1 at DNA level**	Genomic alteration	amplifications		Upregulation	Hodgkin’s lymphoma, SCLC, NSCLC, EBV-positive gastric cancer, and squamous cell carcinoma of the oral cavity.	[20–23, 25, 26]
		translocations		Upregulation	mediastinal large B cell lymphoma	[24]
	Epigenetic regulation	histone deacetylases	HDAC3	Upregulation	drug-resistant cancer	[27]
			HDAC6	Upregulation	melanoma	[28]
		methylation of DNA at CpG sites		Downregulation	melanoma, NSCLC, HNSCC, and CRC	[29–32]
		H3K4me3		Upregulation	pancreatic cancer and breast cancer	[33, 34]
		H3K27me3		Downregulation	HCC	[35]
**Regulation of PD-L1 at RNA level**	Inflammatory signaling	IFN-γ		Upregulation	sarcoma, colon cancer, melanoma, and NSCLC	[36–38]
		IFN-α, IFN-β		Upregulation	melanoma	[39]
		TLR4		Upregulation	bladder cancer	[40, 41]
		TLR3		Upregulation	neuroblastoma	[42]
		IL-17		Upregulation	prostate cancer and colon cancer	[43]
		IL-4		Upregulation	RCC	[44]
		IL-27		Upregulation	ovarian cancer; Prostate cancer; NSCLC	[45]
		IL-6		Upregulation	prostate cancer; HCC; GBM; NSCLC	[46–49]
		IL-10		Upregulation	OSCC	[50]
		TGF-β		Upregulation	NSCLC	[51, 52]
	Aberrant oncogenic signaling	EGFR			NSCLC and LUAD	[53, 54]
		ERK			multiple myeloma, breast cancer, NSCLC, bladder cancer, pancreatic cancer, and lymphoma	[55–60]
		PTEN		Downregulation	breast cancer	[58, 61–69]
		PTEN/PI3K/AKT		Upregulation	gastric cancer, NSCLC, CRC, glioma, melanoma, HNSCC, CRC, and Her2- amplified cancer	[70]
		JAK-STAT		Upregulation	breast cancer and NSCLC	[23, 71, 72]
		NF-κB		Upregulation	NSCLC, breast cancer, melanoma	[73–77]
		HIF-1		Upregulation	LUAD, RCC	[78–84]
		MYC		Upregulation	melanoma, NSCLC, ESCC, leukemia, lymphoma, and pancreatic cancer	[85–92]
				Downregulation	HCC	[93]
		ALK		Upregulation	NSCLC, lymphoma, LUAD	[79, 94–97]
		Met		Upregulation	NSCLC	[98–100]
		BRD4		Upregulation	ovarian cancer, lymphoma	[101, 102]
		DSB		Upregulation	osteosarcoma, NSCLC, and prostate cancer	[103]
		AP-1		Upregulation	Hodgkin’s lymphoma	[104, 105]
		SHH		Upregulation	gastric cancer	[106]
		P53		Upregulation	melanoma, NSCLC, mesothelioma, GBM	[107–110]
	Epigenetic regulation	N6-methyladenosine (m6A) methylation		Downregulation	HNSCC, LUAD, and colon cancer	[111–114]
	Direct regulation by miRNA	miR-34a		Downregulation	AML and lymphoma	[115, 116]
		miR-142-5p		Downregulation	pancreatic cancer	[117]
		miR-93, miR-106b		Downregulation	pancreatic cancer	[118]
		miR-138-5p		Downregulation	CRC	[119]
		miR-217		Downregulation	laryngeal cancer	[120]
		miR-200		Downregulation	NSCLC and gastric cancer	[121, 122]
		miR-152		Downregulation	gastric cancer	[122]
		miR-570		Downregulation	gastric cancer	[123]
		miR-17-5p		Downregulation	melanoma	[124]
		miR-15a, miR-193a, miR-16		Downregulation	malignant pleural mesothelioma	[125]
		miR-148a-3p		Downregulation	CRC	[126]
		miR-873		Downregulation	breast cancer	[127]
		miR-424(322)		Downregulation	ovarian cancer	[128]
		miR-214		Downregulation	lymphoma	[129]
		miR-497-5p		Downregulation	RCC	[130]
		miR-140		Downregulation	NSCLC	[131]
	Indirect regulation by miRNA	miR-20, miR-21, miR-130b		Upregulation	CRC	[132]
		miR-23a-3p		Upregulation	HCC	[133]
		miR-18a		Upregulation	cervical cancer	[134]
		miR-197		Downregulation	NSCLC	[135]
		miR-145		Downregulation	ovarian cancer	[136]
	mRNA stablization	RAS		Upregulation	RAS mutant cancer	[137]
		Angiotensin II		Upregulation	NSCLC	[138]
		G3BP2		Upregulation	breast cancer and GBM	[139]
**Regulation of PD-L1 at protein level**	Ubiquitination	c-Cbl and Cbl-b		Downregulation	NSCLC	[140]
		USP22		Upregulation	HCC	[141]
		USP9X		Upregulation	OSCC	[142]
		UCHL1		Upregulation	NSCLC	[143]
		CMTM6		Downregulation	melanoma, NSCLC, CRC, thyroid cancer, pancreatic cancer, breast cancer	[144, 145]
		CMTM4		Downregulation	NSCLC and melanoma	[145]
		CDK4		Downregulation	cervical cancer and breast cancer	[146]
		CSN5		Downregulation	breast cancer	[147]
	Phosphorylation	Tyr112		Upregulation	HCC	[148]
		S195		Downregulation	breast cancer	[149]
		GSK3β		Downregulation	breast cancer	[150, 151]
	Glycosylation	N192/200/219		Upregulation	breast cancer	[150]
		STT3		Upregulation	cancer stem cell	[152]
		FKBP51s		Upregulation	glioma	[153]
	Palmitoylation	C272		Upregulation	breast cancer and colon cancer	[154, 155]
**Extracellular PD-L1**	exosome			Upregulation	HNSCC, breast cancer, and melanoma	[156–159]
	soluble protein			Upregulation	NSCLC	[160–163]
**Biomarkers**	TMB			Upregulation	multiple cancer types	[164]
	MSI			Upregulation	multiple cancer types	[165]
	TIL			Upregulation	multiple cancer types	[166]
	Intratumor heterogeneity			Upregulation	multiple cancer types	[167]

### Epigenetic regulations

Histone acetylation and methylation regulate gene transcription epigenetically. Reports indicate that alternation of histone deacetylases (HDACs) expression promotes tumorigenesis [168]. In cancer cells with drug resistance, decreased COP1 enhanced JNK/c-Jun signaling activation, consequently inhibiting histone deacetylase 3 (HDAC3) expression. Subsequently, the enhanced histone H3 acetylation of the CD274 promoter upregulated PD-L1 expression [27]. As another HDAC family member, Histone deacetylase 6 (HDAC6) regulates histone acetylation by targeting nonhistone substrates. HDAC6 causes STAT3 phosphorylation, where HDAC6 and phospho-STAT3 are then recruited to the CD274 promoter, mediating the upregulation of PD-L1 in melanoma [28]. Additionally, HDAC inhibitors downregulate PD-L1 expression in melanoma [169], liver cancer [170], anaplastic thyroid cancer [171], NSCLC [172] and triple-negative breast cancer [173] which subsequently enhance and potentiate immunotherapy response. These findings collectively validate the use of HDAC inhibitors combined with PD-L1 inhibitors as a novel therapeutic option.

As the most common epigenetic changes in cancer, DNA hypomethylation at CpG sites enhances PD-L1 expression [174]. Accumulating evidence confirms that CD274 promoter methylation determines PD-L1 expression and predicts the survival of melanoma patients, NSCLC, head, and neck squamous cell carcinomas (HNSCC) CRC [29–32]. DNA methyltransferase inhibitors (DNMTis) were associated with elevated expression of PD-L1 in epithelial cancer cells [175, 176]. DNMTis was proposed to increase DNA hypermethylated endogenous retroviruses (ERVs) expression, which presumably stimulated IFN genes (STING) and generated IFN-γ-induced PD-L1 [174]. The upstream signaling protein of DNMTis, enhancer of zeste two polycomb repressive complex two subunits (EZH2), downregulated PD-L1 expression in hepatocellular carcinoma (HCC) [35]. TGF-β1 inactivated DNMTs and demethylated CD274 promoter in NSCLC positively regulated PD-L1 expression [177], whereas TNF-α activated the nuclear factor kappa-light-chain-enhancer of activated B cells (NF-kB) pathway that demethylated CD274 promoter and promoted PD-L1 expression [177]. A recent study demonstrated that anti-PD-1 therapy triggered more hypermethylated promoters of CD274 in the secondary gastric cardia adenocarcinoma (GCA) compared to the primary GCA without anti-PD-1 therapy [178]. Besides, DNA hypomethylating agent azacytidine combined with anti-PD-1 exhibited a better suppressing capability of tumor growth than anti-PD-1 therapy alone in the mice model [178].

H3K4me3 is an 'activating' histone modifier in reflecting gene transcription [179] and enriched with CD274 promoter in pancreatic cancer. The binding protein of the CD274 promoter, MLL1, catalyzes H3K4me3 to promote PD-L1 transcription [33]. The combined inhibition of MLL1 and anti-PD-1 immunotherapy significantly and synergistically suppresses pancreatic tumor growth [33]. Furthermore, H3K4me3 is actively implicated in epithelial to mesenchymal transition (EMT)-induced PD-L1 expression in breast cancer stem cells [34]. In contrast, H3K27me3, upregulated by EZH2, suppressed PD-L1 expression in HCC [35]. Additionally, epigenetic modulators targeting EZH2-mediated H3K27me3 enhances the efficacy of anti-PD-1 therapy [180].

HDAC domain mutation in transcription factor Tcf1 promotes the CTLA-4 expression in T follicular helper (TFH) cells [181]. Also, methylation of DNA at CpG sites epigenetically suppressed CTLA-4 expression [182].

## Regulation of PD-L1 and CTLA-4 at RNA level

### Inflammatory signaling

Evidence suggests that PD-1/ PD-L1 axis restrains T cell hyperactivity. Consistently, inflammatory signaling regulates the expression of PD-L1. Among the multiple soluble inflammatory factors, type II interferon, IFN-γ, primarily promotes PD-L1 production [183]. The blockage of IFN-γ in the sarcoma mouse model tremendously abrogated PD-L1 expression [36]. In addition to diverse cancer types, IFN-γ-induced PD-L1 is extended in healthy tissues and immune cells [4]. As a pro-inflammatory cytokine, IFN-γ is largely produced by T and NK cells. Subsequently, IFN-γ binds to its receptor, interferon-gamma receptor (IFNGR), and activates the Janus kinase signal transducer and activator of transcription (JAK-STAT) signaling pathway via STAT1. Consequently, it upregulates expression of transcription factors, preferentially the interferon-responsive factors (IRFs) [39, 184]. As the vital downstream signaling protein of STAT1, IRF1 critically mediates IFN-γ-induced PD-L1 [185]. IRF1-deficient mouse models in colon cancer and melanoma showed inhibited tumor growth and upregulation of PD-L1 expression [37]. Moreover, PD-L1 expression is highly correlated with IRF1 in lung cancer [38]. IFN-γ-induced accumulation of IRF1 saturates STXBP6 and stimulates nuclear translocation of IRF1 that inhibits STXBP6 expression and triggers the migration of more IRF1 to the nucleus; this positive feedback loop regulates PD-L1 transcription [186]. IRE1/2 constitutes the binding sites of IRF1 in the CD274 promoter, regulating PD-L1 transcription in HCC [187]. Furthermore, as a major component of the AK-STAT-IRF1 pathway, JAK1 and JAK2 mutations downregulate PD-L1 expression and reduce the efficacy of anti-PD-1 therapy [188]. Therefore, besides PD-1/ PD-L1 axis, tumor cells might develop alternative escaping immune surveillance approaches [188].

In addition to IFN-γ, type I interferons, IFN-α, IFN-β could stimulate PD-L1 expression. Notably, type I interferons regulate PD-L2 expression compared to PD-L1 expression in melanoma [39]. Lipopolysaccharide (LPS) activates toll-like receptor (TLR) 4-nuclear factor kappa-light-chain-enhancer of activated B cells (NF-κB)-IFN-γ signaling cascade and subsequently induces PD-L1 expression in bladder cancer [40, 41]. Similarly, polyinosinic-polycytidylic acid (poly (I: C))-stimulated TLR3 upregulates the expression of PD-L1 in neuroblastoma [4, 42].

Other inflammatory stimuli including IL-1b, IL-4, IL-6, IL-10, IL-12, IL-17, IL-27, tumor necrosis factor-α (TNF-α), and transforming growth factor-β (TGF-β) have also been linked to PD-L1 induction. For instance, IL-4 and TNF-α exert synergistic effects on the PD-L1 induction in renal cell carcinoma (RCC) by activating signaling molecules, including NF-κB, IκB, and STAT6 [44]. Blockade of PD-L1 was accompanied by a downregulation of IL-10 in dendritic cells and monocytes [189]. Besides, the IL-10 abundance directly correlated with PD-L1 expression on TAMs in OSCC [50]. IL-12 demonstrated a dual impact on PD-L1 regulation, i.e., upregulating PD-L1 expression in monocyte-derived macrophages, whereas downregulating PD-L1 expression in THP-1-derived macrophages [190]. IL-17 mediates PD-L1 induction in monocytes [191]. IL-17 and TNF-α individually upregulate PD-L1 expression by activating NF-κB signaling in prostate cancer, as well as NF-κB and ERK1/2 signaling in colon cancer [43]. IL-1b and IL-27 upregulate the expression of PD-L1 in dendritic cells [192]. Moreover, IL-27 promotes PD-L1 expression via phospho-STAT1 and phospho-STAT3 [45].

IL-6-triggered JAK-STAT3 signaling pathway upregulated PD-L1 expression in prostate cancer, which IL-6-expressing tumor is resistant to NK cell-mediated immune action [46]. Besides, IL-6 induces activation of JAK2/STAT3/c-MYC signaling cascade that enhances miR-25-3p transcription and suppresses protein tyrosine phosphatase receptor type O (PTPRO), where the depletion of PTPRO further causes PD-L1 secretion by impairing the activity of JAK2/STAT1 and JAK2/STAT3/c-MYC signaling pathways [47]. Additionally, glioblastoma (GBM)-derived IL-6/STAT3 signaling pathway correlated with myeloid PD-L1 expression [48]. IL-6-MEK/ERK signaling pathway promoted the up-regulation of PD-L1 expression, causing an immune escape in lung cancer [49]. Previous studies demonstrated that HCC-CAF-derived IL6 activates the STAT3-PDL1 signaling cascade [193]. Further, IL6 blockade upregulated PD-L1 expression in melanoma, where the IL6-PD-L1 axis was described as a rational immunosuppressive target for anti-PD-L1 therapy [194].

TGF-β positively regulates the expression of PD-L1. Tumor-infiltrating CD8+ T cells produced TGF-β and subsequently upregulated PD-L1 expression [195]. TGF-β upregulated PD-L1 expression in dendritic cells of lung cancer [51]. Further, TGF-β upregulated PD-L1 expression via Smad-binding elements in NSCLC cells [52]. Nevertheless, several studies revealed that TGF-β repressed PD-L1 expression in monocytes or proximal renal tubular epithelial cells [196, 197].

### Aberrant oncogenic signaling

Oncogenic signaling pathways regulate tumor cell survival, proliferation, and progression. Accumulating evidence indicates that certain oncogenic signaling pathways promote PD-L1 expression.

### Epidermal Growth Factor Receptor (EGFR)

EGFR signaling regulates tumor proliferation, progression, angiogenesis, evasion of apoptosis, and evasion of immunity [198]. EGFR mutation in epithelial cells of NSCLC induced PD-L1 expression, where EGFR inhibitors significantly lowered PD-L1 expression in NSCLC with activated EGFR. Moreover, anti-PD-1 therapy enhanced effector T-cell function and suppressed tumor proliferation in EGFR-driven LUAD [53]. These findings indicate that EGFR signaling caused immune escape. A retrospective study reported a conflicting result, i.e., concurrent upregulation of PD-L1 expression and tumor-infiltrating CD8+ T cells were rarely observed in EGFR-driven NSCLC. Besides, NSCLC with EGFR mutations was resistant to PD-L1 inhibitors [54]. Notably, NSCLC patients receiving standard EGFR-tyrosine kinase inhibitors (TKIs) treatment showed downregulated EGFR-induced PD-L1 expression and upregulated IFN-γ-induced PD-L1 expression. Summarily, PD-L1 expression was dynamically regulated by diverse factors [199].

### Extracellular signal-regulated kinase (ERK)

Mitogen-activated protein kinase (MAPK), also termed extracellular signal-regulated kinase (ERK), promotes cancer development in multiple cancer types [200]. Previous studies confirmed that PMA-activated or variant of MEK (MEK-DD)-activated MEK-ERK signaling regulate PD-L1 expression via IFN-γ-induced STAT1 phosphorylation in multiple myeloma and triple-negative breast cancer (TNBC) [55, 56]. Similarly, in NSCLC, activation of the MAPK signaling pathway was inferred by RAS or MEK activation positively correlated with CD274 transcriptional level [57]. CD274 gene expression was also upregulated by activation of EGF-MAPK signaling [57]. MAPK signaling primarily targets JUN that subsequently cooperates with STAT3 upregulating PD-L1 expression in BRAF inhibition (BRAFi)-resistant melanoma and NSCLC, where the signaling cascade is reversible by MEK inhibition (MEKi) [58, 59].

Further, p38-MAPK, another MAPK signaling cascade, has a positive regulation on PD-L1 expression in dendritic cells, multiple myeloma, bladder cancer, and lymphoma [4]. Notably, MEKi could abrogate the upregulated PD-L1 expression stimulated by TLR ligands [4]. Furthermore, the EGFR-MAPK signaling cascade, activated by myeloid cells, could upregulate PD-L1 expression in pancreatic cancer [60].

### PTEN/PI3K/AKT signaling pathway

PTEN regulates suppressing PI3K-AKT signaling cascade in multiple cancer types [201]. In TNBC expressing PD-L1, PTEN deficiency was frequently observed, and its knockdown upregulated the expression of PD-L1 [70]. In addition, the activated PI3K-AKT signaling pathway caused an upregulated expression of PD-L1 in gastric cancer [61]. Moreover, the PI3K-AKT signaling pathway upregulated IFN-γ-stimulated PD-L1 expression in NSCLC, CRC, glioma, breast cancer, and melanoma [62–66], while PI3K inhibition downregulated PD-L1 expression in HNSCC cells, CRC cells, melanoma cells, and Her2-amplified cancer cells [58, 67–69]. Besides, as a vital downstream of the PI3K-AKT signaling pathway, mTOR can be inhibited by rapamycin to lower the expression of PD-L1 in NSCLC [63].

Specifically, miR-20, miR-21, and miR-130b enhanced PD-L1 expression in CRC by targeting PTEN [132]. miR-23a-3p promoted PD-L1 expression in HCC-derived macrophages by repressing PTEN [133]. Similarly, miR-18a upregulated PD-L1 expression by targeting PTEN, WNK2, and SOX6, which activates PI3K/AKT, MEK/ERK Wnt/β-catenin signaling pathways as well as the inhibitors of the p53 signaling pathway in cervical cancer [134].

### JAK-STAT signaling pathway

JAK-STAT signaling pathway mediates most anti-tumor immune responses, including tumor cell identification and tumor-driven immune evasion [202]. As mentioned in the inflammatory signaling, STAT3 phosphorylation enhanced PD-L1 transcription while STAT inhibition hampered PD-L1 transcription. A novel STAT3 mutation, p.E616K triggered a robust binding of STAT3 to PD-L1 promoter and subsequently increased PD-L1 transcription [203]. Moreover, JAK and STAT3 amplification enhanced PD-L1 expression in TNBC and NSCLC [23, 71]. Ataxia Telangiectasia Mutated (ATM) upregulated PD-L1 expression through activated JAK/STAT3 signaling pathway in lung cancer with cisplatin resistance [72]. Besides, miR-197 downregulated PD-L1 expression by directly activating the CKS1B-STAT3 signaling cascade in NSCLC [135].

### NF-κB family

NF-κB cooperates with multiple oncogenic signaling pathways during cancer initiation and progression [204] and is increasingly related to high PD-L1 expression [73, 177, 205–207]. Immune escape was caused by activation of the NF-κB-PD-L1 axis [208]. The overexpression of MUC1-C caused an increased binding of NF-κB p65 to CD274 promoter and subsequently increased PD-L1 transcription in NSCLC [74]. Likewise, the stimulator of IFN genes (STING)/TANK-binding kinase-1 (TBK1) signaling pathway recruit NF-κB p65 to CD274 promoter and activates both NF-κB and IRF3, contributing to PD-L1 transcription [75]. NF-κB also drives ZEB1 expression, which serves as a transcription factor that mediates EMT [209]. The NF-κB-stimulated ZEB1 expression further suppressed miR-200-induced differentiation of EMT and enhanced PD-L1 expression [76]. Accumulated reactive oxygen species (ROS) activated NF-κB signaling in triple-negative breast cancer [77]. Additionally, inhibited NF-kB pathway suppresses IFN-γ-induced PD-L1 expression in melanoma cells [73].

### Hypoxia-inducible factor-1 (HIF-1)

Hypoxia creates a permissive tumor microenvironment (TME) that facilitates tumor metastasis and immune escape [210]. The cellular colocalization of HIF-1α and PD-L1 was observed in various cancer cell types [78]. The hypoxia-induced PD-L1 expression was suppressed after HIF-1α was blocked using glyceryl trinitrate (GTN) [78]. Besides, HIF-1α regulated PD-L1 expression in LUAD and myeloid-derived suppressor cells (MDSCs) [79, 80]. HIF-1α could also activate NF-κB signaling by inducing transcription factor IKKβ through the hypoxia response element (HRE) [81]. In turn, NF-κB activated HIF-1α by directly binding to the HIF-1α promoter, which the reinforced NF-κB and HIF-1α mutually sustained PD-L1 expression [82]. Moreover, EZH2 upregulates PD-L1 expression through a HIF-1α-dependent manner [83]. HIF-2α also interacts with the HRE in the *PD-L1* promoter and regulates *PD-L1* expression in RCC [84].

### MYC

Overexpressed oncogene, MYC, is frequently observed in most human cancers and promotes tumorigenesis [211]. Inactivation of MYC downregulated *PD-L1* expression in melanoma, NSCLC, esophageal squamous cell carcinoma (ESCC), leukemia, lymphoma, and pancreatic cancer [85–90]. Furthermore, chromatin immunoprecipitation (ChIP)-seq assay revealed that *MYC* binds to *PD-L1* promoter [86], indicating that MYC directly regulates PD-L1 expression transcriptionally. Cyclin-dependent kinase 7 (CDK7)/MYC/PD-L1 signaling cascade have been demonstrated to enhance PD-L1 expression, and the CDK7 inhibitor, THZ1, downregulated PD-L1 expression by suppressing MYC activity in NSCLC [91]. MYC also critically mediated S100A9-induced PD-L1 expression [92]. In contrast, *MYC* negatively regulated IFN-γ-induced *PD-L1* expression in HCC [93]. In addition, MYC inhibition hampered tumor growth, enhanced tumor immune cell infiltration, upregulated *PD-L1* expression, and sensitized anti-PD1 immune response [212]. Additionally, miR-145 downregulated the expression of *PD-L1* by repressing *c- MYC* in ovarian cancer [136].

### Bromodomain-containing protein 4 (BRD4)

As an essential member of the bromodomain and extra terminal (BET) family, BRD4 is known in cancer as a super-enhancer and regulator of oncogenes [213]. BRD4 directly targets CD274 since the ChIP-seq assay revealed a positive correlation between CD274 promoter and BRD4 in ovarian cancer [101]. Besides, BET inhibition downregulated PD-L1 expression and suppressed tumor progression [101]. BET inhibitor (BETi), JQ1, also limited PD-L1 transcription by decreasing BRD4 occupancy at CD274 promoter without changes in MYC occupancy in lymphoma [102]. Conversely, JQ1 was reported to decrease PD-L1 expression by downregulating MYC [86]. The contradictory role of BETi needs to be further verified.

### Anaplastic lymphoma kinase (ALK)

Oncogenic ALK signaling resulting from chromosomal rearrangements is present in NSCLC [214]. Previous studies confirmed that NPM-ALK fusion protein promoted PD-L1 expression through STAT3 signaling and MEK-ERK signaling in lymphomas [94, 95]. Similarly, in NSCLC, echinoderm microtubule-associated protein-like 4 (EML4)-ALK fusion gene was associated with higher PD-L1 expression. EML4-ALK upregulates PD-L1 expression as well as activates downstream mediators, ERK, and AKT in NSCLC [79], STAT3, and HIF-1α in LUAD [96]. Besides, ALK correlated positively with PD-L1 expression in lymphoma [97].

### Met

Met oncogene initiates tumor transformation, fosters tumor growth, and modulates the self-renewal ability of cancer stem cells [215]. In NSCLC, amplification of Met was positively related to PD-L1 expression [98, 99]. Met suppression using Met inhibitors or Met activation using hepatocyte growth factor (HGF) upregulated or downregulated PD-L1 expression in different human cancer cell lines, respectively [100].

### DNA double-strand break (DSB) repair signaling pathway

Exogenous cellular stress stimulates PD-L1 expression in cancer, where DSB represents the most vital type of genotoxic stress. The upregulated PD-L1 expression in osteosarcoma, lung cancer, and prostate cancer in response to DSB required the activation of STAT1/3–IRF1 signaling pathway by ATM/ATR/Chk1 kinases [103]. Moreover, depleted BRCA2 or Ku80 enhanced PD-L1 expression in the X-rays-induced DSB signaling cascade [103].

### Activator protein-1 (AP-1)

As a dimeric transcription factor, AP-1 regulates cell life and death and was recently identified as a potent oncogene [216]. AP-1 containing PD-L1 enhancer element increased PD-L1 promoter activity and elevated PD-L1 transcription in Hodgkin's lymphoma [104]. EBV-encoded latent membrane protein-1 (LMP1) previously triggered AP-1 activity in lymphoma [105]. In line with this finding, EBV-infected lymphoproliferative cancer was detected with high PD-L1 expression [104].

### Sonic hedgehog (SHH) signaling pathway

The SHH signaling pathway is reactivated in gastric cancer and induces PD-L1 expression [217]. *H. pylori* significantly stimulated PD-L1 expression by mediating SHH signaling pathway in gastric cancer [106]. In a clinical trial of basal cell carcinoma, patients experiencing SHH pathway-directed therapy exhibited a better response to anti-PD-1 therapy [218].

### P53

P53 is another classical oncogene and therapeutic target in cancer [219] regulating IFN-γ-stimulated PD-L1 expression in melanoma by JAK2 overexpression [107]. p53 regulates PDL1 expression via miR-34 in NSCLC [108] and via miR-320a in mesothelioma [109]. Notably, nanomedicine with p53 significantly upregulated PDL1 expression in GBM [110].

### Epigenetic regulation

N6-methyladenosine (m6A) methylation is the most common mRNA modification and reportedly regulates carcinogenesis. Based on the large-scale bioinformatics analysis, m6A regulators correlated with PD-L1 expression in HNSCC and lung adenocarcinoma (LUAD) [111, 112]. m6A-binding protein YTHDF1 mediates m6A methylation, where the therapeutic efficacy of anti-PD-1 therapy is enhanced in YTHDF1-knockout mice [113]. Fat mass and obesity-associated protein (FTO) demethylates m6A, depleting FTO downregulated IFN-γ-induced PD-L1 expression in colon cancer [114].

### Regulation by microRNA (miRNA)

miRNAs physiologically function as post-transcriptional regulators for gene expression [220], where miRNAs also regulate PD-L1 expression by binding to CD274 mRNA or affecting PD-L1 regulators. miR-513, suppressed by IFN-γ, directly binds to the 3' UTR of CD274 and suppresses PD-L1 expression [221]. Similarly, miR-513, enforced by TNF-α and IFN-γ, binds to the 3' UTR of CD274 and suppresses PD-L1 expression [222]. miR-34a also binds to 3' UTR of CD274 and lowers PD-L1 expression in acute myeloid leukemia (AML) and lymphoma [115, 116]. Moreover, miR-142-5p [117], miR-93, and miR-106b [118] regulated PD-L1 expression in pancreatic cancer. miR-138-5p regulated PD-L1 expression in CRC [119]. miR-217 regulated PD-L1 expression in laryngeal cancer [120]. miR-200 regulated PD-L1 expression in NSCLC and gastric cancer [121, 122]. miR-152 [122] and miR-570 [123] regulated PD-L1 expression in gastric cancer. miR-17-5p regulated PD-L1 expression in melanoma [124]. miR-15a, miR-193a, and miR-16 regulated PD-L1 expression in malignant pleural mesothelioma [125]. miR-148a-3p regulated PD-L1 expression in CRC [126]. miR-873 regulated PD-L1 expression in breast cancer [127]. miR-424 (322) regulated PD-L1 expression in ovarian cancer [128]. miR-214 regulated PD-L1 expression in lymphoma [129]. miR-497-5p regulated PD-L1 expression in RCC [130]. miR-140 regulated PD-L1 expression in NSCLC [131]. These miRNAs have all been identified as critical suppressors of PD-L1 expression.

Notably, post-transcriptional regulators miRNAs also regulated CTLA-4 expression; for example, miR-155 increased the T helper (TH) cell proliferation through downregulating CTLA-4 expression [223]. circRNA-0003528 promoted CTLA-4 expression downregulating miR-224-5p, miR-324-5p and miR-488-5p^193^. Moreover, 3'-UTR variation affected the repressive ability of miR-302a in inhibiting CTLA-4 expression [224].

### Stability of PD-L1 mRNA

The structural disruption in the 3' untranslated regions (UTR) influences CD274 mRNA stability causing elevated PD-L1 expression [225]. RAS, downstream of MEK signaling, of which activation phosphorylated tristetraprolin (TTP) and inhibited TTP-mediated AU-rich element (ARE)-binding to the 3' UTR of PD-L1 mRNA via kinase MK2, which stabilized CD274 mRNA and consequently elevated PD-L1 expression [137]. Similarly, Angiotensin II stabilized CD274 mRNA and upregulated PD-L1 expression in NSCLC via human antigen R (HuR), another ARE-binding protein [138]. A recent study also confirmed that GTPase-activating protein (SH3 domain)-binding protein 2 (G3BP2) stabilized PD-L1 mRNA through its RNA recognition motif and upregulated PD-L1 expression under stress in breast cancer and GBM [139].

### Transcriptional regulation of CTLA-4

**Fig. 3:**
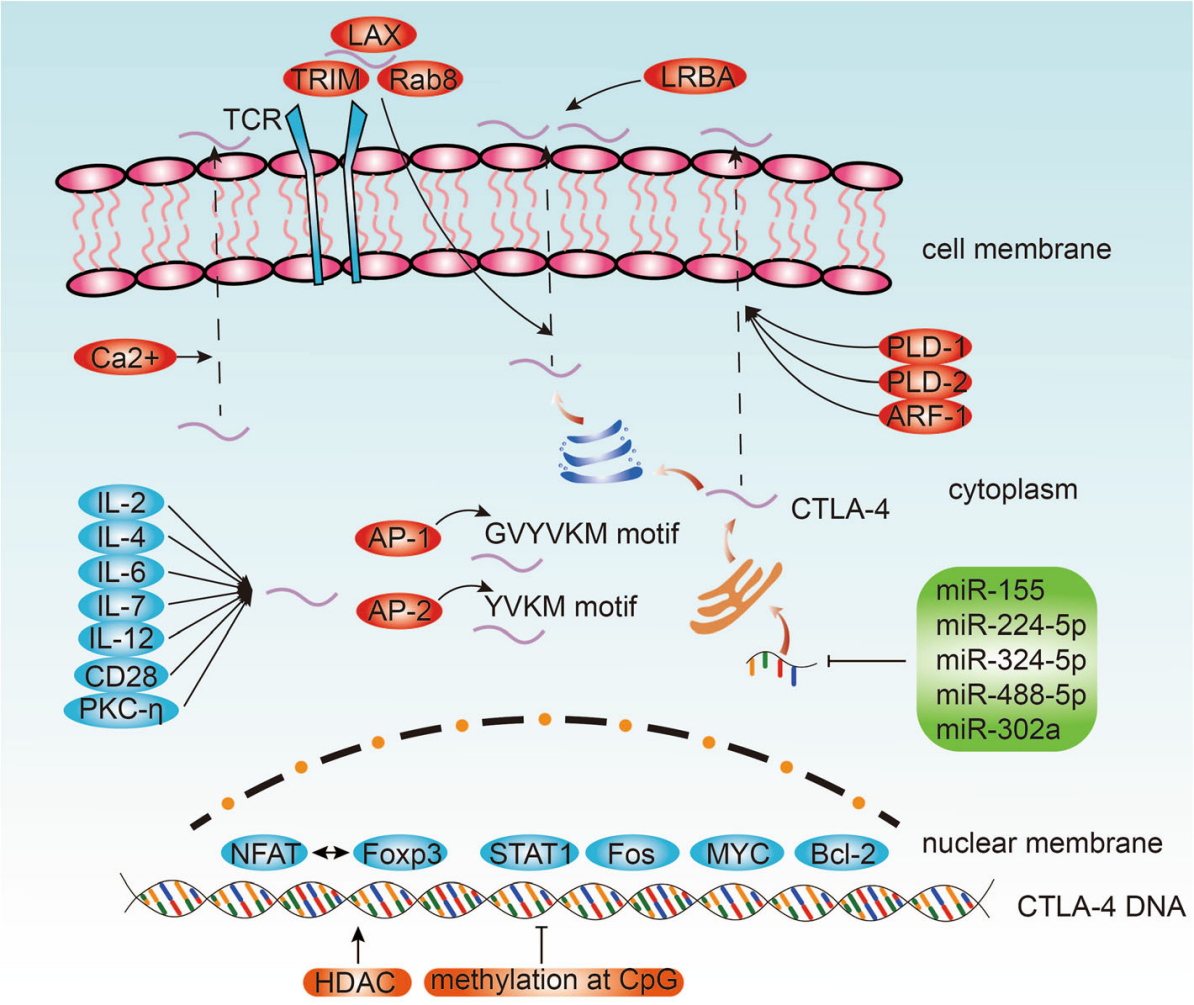
Regulatory mechanisms of CTLA-4. CTLA-4 expression is regulated at RNA level (transcriptional regulation, direct regulation by miRNA) and DNA level (epigenetic regulation). Localization within the cell also regulate CTLA-4 expression.

Studies exploring the transcriptional regulation of CTLA-4 are limited. The nuclear factor of activated T-cells (NFAT) binds to the CTLA-4 promoter and promotes CTLA-4 transcription (Fig. 3, Table 2). Furthermore, the mutated NFAT site abolished the activity of the CTLA-4 promoter, where inhibitors of NFAT also suppressed CTLA-4 transcription [226]. Forkhead box P3 (Foxp3) is another transcription factor interacting with NFAT at CTLA-4 promoter and activated CTLA-4 transcription [227]. The transcriptional activity of Foxp3 was reported be reinforced by transcription factors including GATA-1, Lef1, Eos, IRF4, and Satb1 [242]. CD28 has been recommended for its counteraction with CTLA-4 [228]. Stimulation with IL-1α, IFN-γ, IL-2, and inhibition of CD28 have all been proved to promote CTLA-4 expression [229], while IL-4, IL-6, IL-7, and IL-12 only showed limited ability in inducing CTLA-4 expression [230]. The interaction between CTLA-4 and STAT5 was proved to critically mediate CTLA-4 signaling [231]. STAT1, c-Fos, c-MYC, and Bcl-2 positively correlate with CTLA-4 expression in chronic lymphocytic leukemia (CLL) [232]. Kinase PKC-η also regulated CTLA-4 expression, and the CTLA-4-PKC-η axis was essential for Treg function [233].

**Table 2:** Regulators of CTLA-4

Regulatory Types		Regulators	CTLA-4	References
**Regulation of CTLA-4 at DNA level**	Epigenetic regulation	HDAC	Upregulation	[181]
		methylation of DNA at CpG sites	Downregulation	[182]
**Regulation of CTLA-4 at RNA level**	Transcriptional regulation	NFAT	Upregulation	[226]
		Foxp3	Upregulation	[227]
		CD28	Downregulation	[228]
		IL-1α, IFN-γ	Upregulation	[229]
		IL-2	Upregulation	[230]
		STAT5	Upregulation	[231]
		STAT1, c-Fos, c-MYC, and Bcl-2	Upregulation	[232]
		PKC-η	Upregulation	[233]
	Direct regulation by miRNA	miR-155	Downregulation	[223]
		miR-224-5p, miR-324-5p and miR-488-5p	Downregulation	[234]
		miR-302a	Downregulation	[224]
**Localization within the cell**		AP-1	Upregulation	[235]
		AP-2	Upregulation	[236]
		TCR	Upregulation	[237]
		TRIM	Upregulation	[238, 239]
		GTPases, PLD- 1, PLD-2, and ARF-1	Upregulation	[240]
		LRBA	Upregulation	[241]
		Ca^2+^	Upregulation	[237]

## Regulation of PD-L1 at protein level

Post-translational regulation at the protein level is the eventual step where the gene expression level is modulated. Among the multiple post-translational modulators, ubiquitination, phosphorylation, glycosylation, and palmitoylation regulate PD-L1 expression.

### Ubiquitination

The Casitas B-lineage lymphoma (Cbl) family has three major isoforms, c-Cbl, Cbl-b, and Cbl-c, among which c-Cbl and Cbl-b with a ubiquitin-associated (UBA) domain suppressed PD-L1 expression after inhibiting PI3K/Akt, JAK-STAT, and MEK/Erk signaling pathways in NSCLC [140]. Additionally, ubiquitin-specific peptidase 22 (USP22) in HCC [141], ubiquitin-specific peptidase 9, X-linked (USP9X) in OSCC [142], and ubiquitin C-terminal hydrolase L1 (UCHL1) in NSCLC [143] deubiquitinate and upregulate the PD-L1 expression.

As a ubiquitously expressed protein, CMTM6 binds PD-L1 either at the plasma membrane or in recycling endosomes, repressing lysosome-mediated degradation of PD-L1 and upregulating protein half-time of PD-L1 without affecting the transcription level of PD-L1 [144, 145]. Notably, CMTM4 shares a similar function with CMTM6 [145].

Besides, PD-L1 expression is negatively regulated by cyclin-dependent kinase 4 (CDK4). CDK4 indirectly promotes ubiquitination of PD-L1 by phosphorylating the adaptor protein of Cullin 3 (CUL3), SPOP, an E3 ubiquitin ligase for PD-L1 [146]. Inhibition of CDK4/6 upregulated PD-L1 expression [146]. Moreover, NF-κB p65-induced COP9 signalosome 5 (CSN5) stabilized PD-L1 expression by inhibiting ubiquitination of PD-L1 via TNF-α [147].

### Phosphorylation

IL-6/JAK1 signaling cascade phosphorylates PD-L1 Tyr112 that recruits the endoplasmic reticulum-associated N-glycosyltransferase, STT3A. Subsequently, STT3A catalyzes PD-L1 glycosylation and elevates PD-L1 stability [148]. Moreover, metformin-activated AMP-activated protein kinase (AMPK) phosphorylates PD-L1 S195, which enriches aberrant PD-L1 glycosylation. Subsequently, the translocation of PD-L1 was hampered while the degradation of PD-L1 was enhanced [149]. Besides, glycogen synthase kinase 3β (GSK3β) downregulates PD-L1 expression by inducing phosphorylation-dependent proteasome degradation via β-TrCP [150]. Inhibition of Met-mediated phosphorylation and activation of GSK3B downregulated PD-L1 expression [151].

### Glycosylation

PD-L1 N192/200/219 glycosylation antagonized the formation of the complex comprising PD-L1, GSK3β, and β-TrCP and stabilized PD-L1 [150]. Further, EGF stabilized PD-L1 by inactivating glycosylation-induced GSK3β [150]. Moreover, EMT promoted N-glycosyltransferase STT3 via β-catenin in cancer stem-like cells (CSCs). STT3 induced PD-L1 N-glycosylation, and eventually, EMT /β-catenin/STT3 signaling cascade upregulated PD-L1 expression [152]. Besides, FKBP51s promoted PD-L1 expression by catalyzing glycosylation in gliomas [153].

### Palmitoylation

Palmitoylation at C272 stabilized PD-L1 by blocking the ubiquitination of PD-L1 [154]. Further, DHHC3 catalyzed palmitoylation at C272, suppressing PD-L1 expression and promoting anti-tumor immune response [155].

## Extracellular PD-L1

Besides intracellular interactions between various regulators and PD-L1, PD-L1 expression is also regulated when PD-L1 is secreted as exosomes and soluble proteins at the extracellular level.

### PD-L1 in the form of exosome

Exosomal PD-L1 has been reported in various cancer types. Exosomal PD-L1 suppressed T cell activity and promoted tumor progression in HNSCC [156]. Accordingly, blockade of exosomal PD-L1 secretion enhanced T cell activity [157]. Similarly, bioactive exosomal PD-L1 promoted tumor growth while blockade of exosomal PD-L1 secretion enhanced the response rates of anti-PD-L1 therapy in breast cancer [158]. Additionally, IFN-γ stimulates the secretion of exosomal PD-L1 in melanoma [159]. Notably, exosomal PD-L1 expression varied following the stage of anti-tumor immunity [159].

### PD-L1 in the form of soluble protein

Soluble PD-L1 (sPD-L1) was relatively more enriched in the plasma in NSCLC, correlating with poor prognosis and suppressed T cell activity [160]. Besides, patients with high plasma sPD-L1 exhibited enhanced efficacy of anti-PD-L1 therapy [161]. The secretion of four sPD-L1 with spicing variants increased after IFN-α, IFN-γ, and TNF-α treatment, subsequently suppressing T cell activity [162]. Similarly, the secretion of five PD-L1 with splicing variants was observed in NSCLC, among which sPD-L1 with v229 and v242 were related to poor anti-PD-L1 response [163].

## Biomarkers indirectly regulate PD-L1 expression

Tumor mutation burden (TMB) was positively associated with PD-L1 expression, where patients with high TMB showed improved efficacy of anti-PD-1 therapy [164]. Similarly, microsatellite instability (MSI) positively correlates with PD-L1 expression and predicts a better anti-PD-1 response [165]. Intratumor heterogeneity was also linked to PD-L1 expression [167]. Moreover, TIL abundance was confirmed to correlate with upregulated PD-L1 expression [166].

## Localization of CTLA-4 within the cell

Notably, CTLA-4 is mainly localized in intracellular compartments with the minority of CTLA-4 detected on the cell surface. Therefore, summarizing the mechanisms by which CTLA-4 could be transported to the cell surface is important. Plasma membrane-associated clathrin adaptor protein 1 (AP-1) binds to the GVYVKM motif of CTLA-4 and subsequently maintains a steady level of intracellular CTLA-4 expression [235]. Likewise, after AP-2 binds to the YVKM motif of CTLA-4 or the FVKM motif of mutant CTLA-4, CTLA-4 was more localized in the cytoplasm and less accumulated on the T cell surface [236, 237]. The strength of the T cell receptor (TCR) signaling pathway determined the upregulated CTLA-4 expression at the immunological synapse [237]. Besides, the adaptor T cell receptor-interacting molecule (TRIM) binds to CTLA-4, forming a CTLA-4/TRIM/LAX/Rab8 effector complex, which in turn mediates the augment of CTLA-4 expression on the surface of T cell [238, 239]. The activity of guanosine triphosphatases (GTPases), phospholipase D (PLD)- 1, PLD-2, and ADP ribosylation factor (ARF)-1 were necessary for increased release of CTLA-4 to the surface of the T cell [240]. Lipopolysaccharide-responsive and beige-like anchor protein (LRBA) loss causes CTLA-4 degradation and suppresses CTLA-4 expression [241]. Additionally, CTLA-4 expression on the cell surface was rapidly upregulated via the upregulated intracellular calcium (Ca^2+^) [237].

## Potential drug intervention on PD-L1 and CTLA-4

**Fig. 4:**
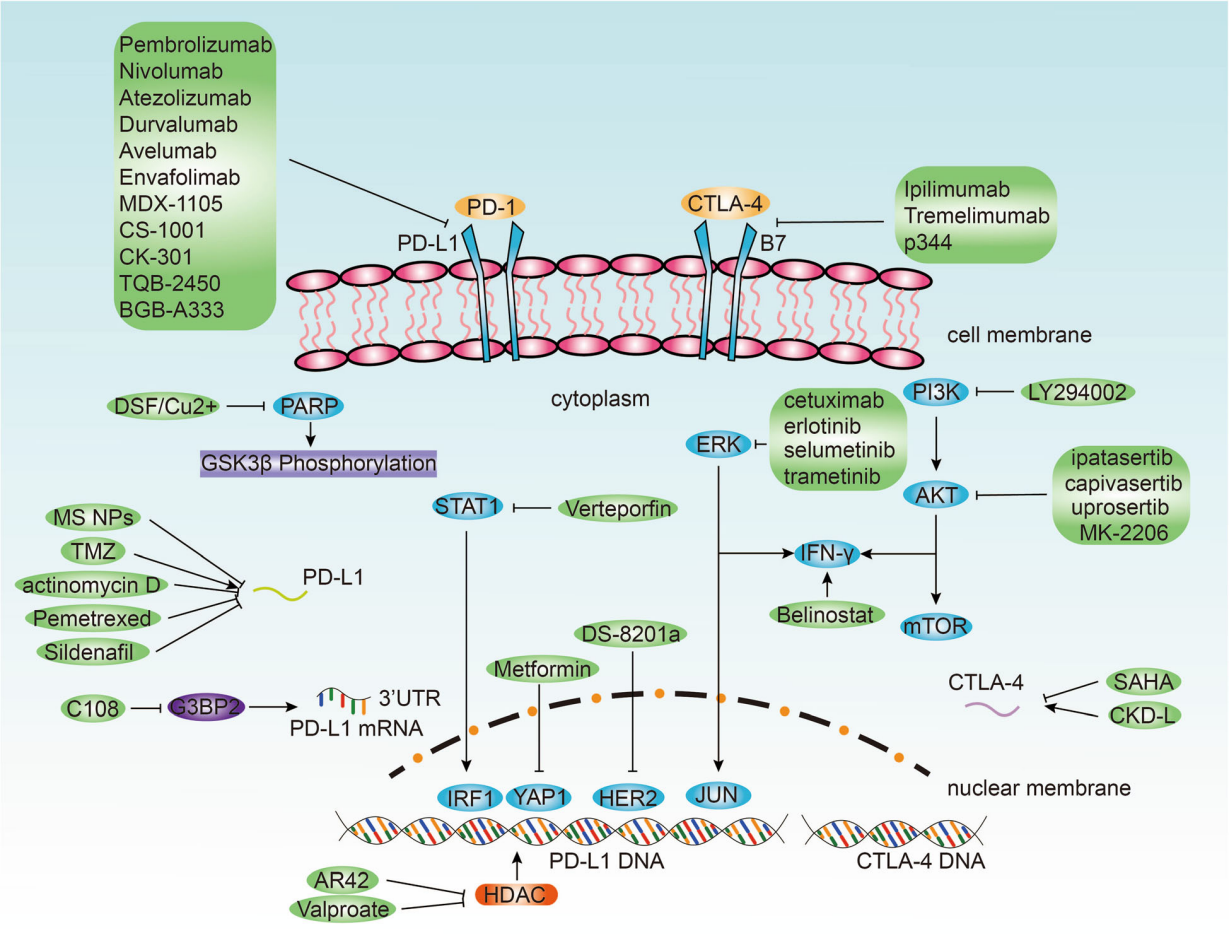
Potential drug intervention on PD-L1 and CTLA-4.

As for PD-L1, multiple drugs exhibited efficacy in regulating PD-L1 expression (Fig. 4, Table 3). Metformin attenuated PD-L1 expression by phosphorylating oncogene YAP1 and inhibiting its entry in the nucleus, subsequently inducing the Hippo signaling pathway in CRC [243]. Further, Nanodrug (MS NPs), self-assembled from metformin and anticancer agent 7-ethyl-10-hydroxycamptothecin (SN38), was developed to suppress PD-L1 expression, enhancing the anti-tumor effect of combined immunotherapy and chemotherapy in breast cancer [244]. PARP inhibitor (PARPi) upregulated PD-L1 expression by inactivating GSK3β and enhancing immunosuppression in breast cancer [245]. Temozolomide (TMZ)-treated GBM was observed with upregulated PD-L1 expression [246]. Further, after the TMZ challenge, actinomycin D suppressed PD-L1 expression in GBM [246]. Pemetrexed and sildenafil suppressed PD-L1 expression in NSCLC, where the inhibitory effect was enhanced by histone deacetylase inhibitors AR42 and sodium valproate [247]. Notably, AR42 and valproate alone downregulated PD-L1 expression [247]. Disulfiram combined with Copper (DSF/Cu^2+^) upregulated PD-L1 expression by inactivating PARP1 and phosphorylating GSK3βat Ser9 in HCC [248]. DSF/Cu^2+^ also inhibited T cell infiltration while failed to hamper HCC tumor growth [248]. The Akt inhibitors ipatasertib, capivasertib, uprosertib, and MK-2206 inhibit PD-L1 expression in breast cancer [249]. Trastuzumab deruxtecan (DS-8201a), a HER2-targeting drug, enhanced PD-L1 expression and CD8+ T cell infiltration in breast cancer, which DS-8201a also had synergistic effect with anti-PD-1 therapy [250]. Verteporfin has been explored as a potential inhibitor that hampers IFN-γ-derived PD-L1 expression by disrupting the STAT1-IRF1-TRIM28 signaling cascade T-cell leukemia, B-cell leukemia, ovarian, osteoblastoma, and NSCLC [251]. PI3K inhibitor (LY294002) downregulated PD-L1 expression in gastric cancer [61]. EGFR inhibitors (cetuximab and erlotinib) or MEK inhibitor (selumetinib) suppressed MAPK signaling-mediated EGF-induced and IFN-γ-induced PD-L1 expression in LUAD [57]. In contrast, MEK inhibitor (trametinib), suppressing MAPK signaling, enhanced the IFN-γ-stimulated PD-L1 expression in breast cancer [56]. Besides, the insignificant alterations and IFN-γ-stimulated PD-L1 expression in diverse cancer cell lines after MAPK inhibitor treatment shed light on the individual regulatory role of PD-L1 in various cancer types [62, 260]. Belinostat enhanced IFN-γ production and upregulated PD-L1 expression in HCC, which Belinostat also had a synergistic effect of anti-PD-1 therapy [252]. C108 repressed G3BP2 and downregulated PD-L1 expression in breast cancer and GBM [139].

**Table 3:** Potential drugs intervening PD-L1 and CTLA-4

Drugs		Mechanism	PD-L1 or CTLA-4	Cancer types	References
PD-L1	metformin	phosphorylating oncogene YAP1	Downregulation	CRC	[243]
	MS NPs		Downregulation	breast cancer	[244]
	PARPi	inactivating GSK3β	Upregulation	breast cancer	[245]
	TMZ		Upregulation	GBM	[246]
	actinomycin D		Downregulation	GBM	[246]
	Pemetrexed and sildenafil		Downregulation	NSCLC	[247]
	AR42 and valproate	inhibiting histone deacetylase	Downregulation	NSCLC	[247]
	DSF/Cu^2+^	inactivating PARP1 and phosphorylating GSK3βat Ser9	Upregulation	HCC	[248]
	ipatasertib, capivasertib, uprosertib, and MK-2206	inhibiting Akt	Downregulation	breast cancer	[249]
	DS-8201a	targeting HER2	Upregulation	breast cancer	[250]
	Verteporfin	inhibiting STAT1-IRF1-TRIM28 signaling cascade	Downregulation	T-cell leukemia, B-cell leukemia, ovarian, osteoblastoma, and NSCLC	[251]
	LY294002	inhibiting PI3K	Downregulation	gastric cancer	[61]
	cetuximab, erlotinib, and selumetinib	inhibiting MAPK signaling	Downregulation	LUAD	[57]
	trametinib	inhibiting MAPK signaling	Upregulation	breast cancer	[56]
	Belinostat	inducing IFN-γ	Upregulation	HCC	[252]
	C108	Inhibiting G3BP2	Downregulation	GBM	[139]
	Pembrolizumab	inhibiting interaction of PD-L1 with PD-1		urothelial cancer, melanoma, HNSCC, and NSCLC	[253]
	Nivolumab and Atezolizumab	inhibiting interaction of PD-L1 with PD-1		NSCLC, urothelial cancer, RCC, and melanoma	[253]
	Durvalumab and Avelumab	inhibiting interaction of PD-L1 with PD-1		urothelial cancer	[253]
	Envafolimab	inhibiting interaction of PD-L1 with PD-1		prostate cancer, NSCLC, breast cancer, gastrointestinal cancer, melanoma, cervical cancer, HNSCC, bladder cancer, cholangiocarcinoma	[254]
	MDX-1105 and CS-1001	inhibiting interaction of PD-L1 with PD-1		NSCLC, melanoma, RCC	[9]
	CK-301	inhibiting interaction of PD-L1 with PD-1		NSCLC, HNSCC, melanoma, RCC, urothelial cancer, Hodgkin’s lymphoma	[254]
	TQB-2450	inhibiting interaction of PD-L1 with PD-1		melanoma	[254]
	BGB-A333	inhibiting interaction of PD-L1 with PD-1		advanced solid tumor	[254]
CTLA-4	Ipilimumab	inhibiting interaction of CTLA-4 with B7		RCC, prostate cancer, cervical cancer, CRC, NSCLC, gastric cancer, pancreatic cancer, ovarian cancer, urothelial cancer, and melanoma	[255]
	Tremelimumab	inhibiting interaction of CTLA-4 with B7		HCC	[256]
	p344	inhibiting interaction of CTLA-4 with B7			[257]
	SAHA		Downregulation		[258]
	CKD-L		Upregulation		[259]

Among the multiple drugs developed for inhibiting the interaction of PD-L1 with PD-1, Pembrolizumab, Nivolumab, Atezolizumab, Durvalumab, and Avelumab have been approved by FDA. Pembrolizumab showed efficiency in regulating the interaction of PD-L1 with PD-1 in urothelial cancer, melanoma, HNSCC, and NSCLC [253]. Similarly, Nivolumab and Atezolizumab inhibited PD-L1 in NSCLC, urothelial cancer, RCC, and melanoma [253]. Durvalumab and Avelumab inhibited PD-L1 in urothelial cancer [253]. Other PD-L1 inhibitors have also been investigated in many clinical trials. Envafolimab (KN 035 or ASC 22) inhibited the interaction of PD-L1 with PD-1 and exhibited encouraging anti-tumor activity in a phase I clinical trial of advanced solid tumor [254]. Besides, the clinical efficacy and safety of BMS-936559 (MDX-1105) and CS-1001 were explored in a phase I clinical trial of advanced solid tumor [9]. Several drugs are currently undergoing clinical trials. CK-301 showed high binding affinity for PD-L1 and increased IFN-γ in a preclinical research [254]. CBT-502 (TQB-2450) is another inhibitor of PD-L1 with increased IFN-γ in melanoma [254]. Except for inhibiting PD-L1, BGB-A333 inhibited T cell apoptosis and increased T cell proliferation [254].

As for CTLA-4, Ipilimumab, a drug approved by FDA, docked to the MYPPPY motif of CTLA-4 and showed efficacy in inhibiting CTLA-4 in RCC, prostate cancer, cervical cancer, CRC, NSCLC, gastric cancer, pancreatic cancer, ovarian cancer, urothelial cancer, and melanoma [255]. Tremelimumab inhibited CTLA-4 in phase II clinical trials of HCC [256]. A synthetic peptide (p344) was demonstrated to bind to the MYPPPY motif of CTLA-4 and blocked its interaction with B7 ligands [257]. As a HDAC inhibitor (HDACi), suberoylanilide hydroxamic acid (SAHA) synergizes with tacrolimus (FK506) to increase Foxp3 and CTLA-4 expression [258]. Besides, HDAC6 inhibitor, CKD-L, increased CTLA-4 expression in Treg [259].

## Conclusion

Immunotherapies represented by targeting PD-1/ PD-L1 and CTLA-4/B7 pathways have shown remarkable clinical efficacies against various cancer types. However, only a small proportion of patients exhibit favorable responses to anti-PD1-PD-L1 or anti-CTLA-4/B7 therapy. Overexpression of PD-L1 and CTLA-4 are recognized as a vital suppressor of anti-tumor immunity and associated with better therapy response and increased clinical benefit. Given the unprecedented predictive values of PD-L1 and CTLA-4 expression in immunotherapy, investigating the diverse regulators on PD-L1 and CTLA-4 expression that could potentially influence immunotherapy efficacy will contribute to the individualized clinical management of cancer patients.

In this review, the mechanisms of PD-L1 regulation were categorized into genetic alteration, epigenetic regulation, inflammatory signaling, and oncogenic signaling at the levels of DNA, regulation at RNA, regulation at protein, extracellular secretion, indirect regulation by biomarkers, and potential drug intervention. In summary, IFN-γ plays a pivotal role in inducing PD-L1 expression through epigenetic regulation, transcriptional regulation, posttranscriptional regulation, and extracellular secretion. In contrast, there is limited literature on the regulatory mechanisms of CTLA-4, with the available literature indicating that the regulation of CTLA-4 mainly depends on its localization within the cell. Targeting IFN-γ and regulating its localization within the cell could perceivably promote the anti-tumor immunotherapy responses. Some potential drugs targeting the PD-L1 regulators, such as IFN-γ, MAPK, and GSK3β exhibited remarkable synergistic anti-tumor effect with ICIs. Notably, the regulation of PD-L1 and CTLA-4 expressions have some similarities in HDAC, methylation of DNA at CpG sites, IL-2, STAT1, c-MYC at DNA level, and miRNA regulation at RNA level. Developing drugs targeting the mutual regulators of CTLA-4 and PD-L1 is expected to improve the therapeutic efficacy.

Further studies should be conducted to investigate more valuable PD-L1 and CTLA-4 regulators to improve the efficacy of immunotherapy and facilitate individualized cancer treatment. Moreover, some regulators with potential synergistic effect with the current ICIs should be expected to be explored in clinical trials.

## Supplementary Information

**Additional file 1.**

## Data Availability

Not applicable.

## Abbreviations

CTLA-4: Cytotoxic T-lymphocyte–associated antigen 4
PD-1: Programmed death 1
PD-L1: Programmed cell death-ligand 1
ICI: Immune checkpoint inhibitor
CAR: Chimeric antigen receptor
CRC: Colorectal carcinoma
NSCLC: Non-small cell lung cancer
TILs: Tumor-infiltrating lymphocytes
TAMs: Tumor-associated macrophages
DCs: Dendritic cells
Tregs: Regulatory T cells
CAFs: Cancer-associated fibroblasts
SCLC: Small cell lung cancer
EBV: Epstein-barr virus
OSCC: Oral squamous cell carcinoma
HDACs: Histone deacetylases
HNSCC: Head and neck squamous cell carcinomas
DNMTis: DNA methyltransferase inhibitors
ERVs: Endogenous retroviruses
STING: Stimulator of IFN genes
EZH2: Enhancer of zeste 2 polycomb repressive complex 2 subunit
HCC: Hepatocellular carcinoma
NF-kB: Nuclear factor kappa-light-chain-enhancer of activated B cells
GCA: Gastric cardia adenocarcinoma
EMT: Epithelial to mesenchymal transition
TFH: T follicular helper
IFNGR: Interferon-gamma receptor
JAK-STAT: Janus kinase-signal transducer and activator of transcription
IRFs: Interferon-responsive factors
LPS: Lipopolysaccharide
TLR: Toll-like receptor
poly(I:C): Polyinosinic-polycytidylic acid
TNF-α: Tumor necrosis factor-α
TGF-β: Transforming growth factor-β
RCC: Renal cell carcinoma
PTPRO: Protein tyrosine phosphatase receptor type O
GBM: Glioblastoma
EGFR: Epidermal growth factor receptor
ERK: Extracellular signal-regulated kinase
MAPK: Mitogen-activated protein kinase
TNBC: Triple negative breast cancer
BRAFi: BRAF inhibition
MEKi: MEK inhibition
ATM: Ataxia Telangiectasia Mutated
TME: Tumor microenvironment
ESCC: Esophageal squamous cell carcinoma
ALK: Anaplastic lymphoma kinase
HGF: Hepatocyte growth factor
BET: Bromodomain and extraterminal
DSB: DNA double-strand break
AP-1: Activator protein-1
SHH: SONIC hedgehog
LMP1: Latent membrane protein-1
m6A: N6-methyladenosine
LUAD: Lung adenocarcinoma
FTO: Fat mass and obesity-associated protein
AML: Acute myeloid leukemia
UTR: Untranslated regions
G3BP2: GTPase-activating protein (SH3 domain)-binding protein 2
NFAT: Nuclear factor of activated T-cells
Foxp3: Forkhead box P3
CLL: Chronic lymphocytic leukemia
Cbl: CASITAS B-lineage lymphoma
USP22: Ubiquitin-specific peptidase 22
UCHL1: Ubiquitin C-terminal hydrolase L1
CDK4: Cyclin D/cyclin-dependent kinase 4
CSN5: COP9 signalosome 5
AMPK: AMP-activated protein kinase
GSK3β: Glycogen synthase kinase 3β
CSCs: Cancer stem-like cells
TMB: Tumor mutation burden
MSI: Microsatellite instability
AP-1: Plasma membrane-associated clathrin adaptor protein 1
TRIM: T cell receptor-interacting molecule
PLD: Phospholipase D
ARF: ADP ribosylation factor
