# Supplementary material for: Regulatory mechanisms of immune checkpoints PD-L1 and CTLA-4 in cancer
Source: J Exp Clin Cancer Res. 2021 Jun 4;40:184. doi: 10.1186/s13046-021-01987-7 (PMC8178863; doi:10.1186/s13046-021-01987-7)
Supplement: Supplementary file 1 — Additional file 1. [file 13046_2021_1987_MOESM1_ESM.docx]

Table S1. Current therapies with ICIs.

| Targets | Drugs | Cancer types |
| --- | --- | --- |
| LAG-3 | IMP321/Eftilagimod alpha | Metastatic RCC; MBC; Disease-free melanoma; Advanced pancreas cancer; Advanced melanoma; Metastatic breast cancer; Metastatic melanoma; Advanced NSCLC and HNSCC |
|  | Relatlimab /BMS-986016 | Advanced solid tumors; Recurrent glioblastoma; Gastro/esophageal cancer; Virus-associated tumors; Advanced hematologic malignancies; Advanced chordoma; Metastatic melanoma; MSS advanced CRC; MSI-H solid tumors; Advanced RCC; Advanced GC; Advanced NSCLC; Advanced CRC |
|  | LAG525 | TNBC; Advanced solid and hematologic malignancies; Advanced melanoma |
|  | MK-4280 | Hematological malignancies; Advanced NSCLC |
|  | REGN3767 | Advanced cancers |
|  | TSR-033 | Advanced solid tumors |
|  | BI754111 | Advanced NSCLC and HNSCC; Advanced solid tumors |
|  | Sym022 | Advanced solid tumor or lymphomas |
|  | FS118^a^ | Advanced malignancies |
|  | MGD013^b^ | Advanced cancers |
| TIM-3 | TSR-022 | Advanced solid tumors; Liver Cancer |
|  | MBG453 | Advanced malignancies; AML or high risk MDS; GBM |
|  | Sym023 | Advanced solid tumor or lymphomas |
|  | INCAGN2390 | Advanced malignancies |
|  | LY3321367 | Advanced solid tumor |
|  | BMS-986258 | Advanced solid tumor |
|  | SHR-1702 | Advanced solid tumor |
|  | RO7121661^c^ | Advanced solid tumor |
| TIGIT | MK-7684 | Advanced solid tumor |
|  | Etigilimab /OMP-313 M32 | Advanced solid tumor |
|  | Tiragolumab/MTIG7192A/RG-6058 | Advanced solid tumor; Advanced NSCLC |
|  | BMS-986207 | Advanced solid tumor |
|  | AB-154 | Advanced malignancies |
|  | ASP-8374 | Advanced solid tumor |
| VISTA | JNJ-61610588 | Advanced solid tumor |
|  | CA-170^d^ | Advanced solid tumors and lymphomas |
| IDO1 | Indoximod (d-1-MT) | Metastatic breast cancer; Melanoma; Metastatic adenoma of pancreas; Acute myeloid leukemia; GBM, glioma, ependymoma, medulloblastoma; Prostate carcinoma; NSCLC |
|  | INCB024360 | Melanoma; Reproductive tract tumors; Colorectal cancer; Gastric cancer; Pancreatic cancer; NSCLC, urothelial carcinoma |
|  | GDC-0919 | Locally advanced or metastatic solid tumors |
|  | IDO1 peptide | NSCLC; Melanoma |
|  | PF-06840003 | GBM |
|  | BMS986205 | Cervical cancer; DLBCL; SCCHN; Urothelial cancer; Pancreatic cancer; Melanoma; NSCLC |
